# Comparative Pathology of Animal Models for Influenza A Virus Infection

**DOI:** 10.3390/pathogens13010035

**Published:** 2023-12-29

**Authors:** Natalie M. Kirk, Yuying Liang, Hinh Ly

**Affiliations:** Department of Veterinary & Biomedical Sciences, College of Veterinary Medicine, University of Minnesota, Twin Cities, MN 55108, USA; kirk0332@umn.edu (N.M.K.); liangy@umn.edu (Y.L.)

**Keywords:** respiratory pathogens, influenza virus, IAV, IBV, animal models, pathology, pathogenesis

## Abstract

Animal models are essential for studying disease pathogenesis and to test the efficacy and safety of new vaccines and therapeutics. For most diseases, there is no single model that can recapitulate all features of the human condition, so it is vital to understand the advantages and disadvantages of each. The purpose of this review is to describe popular comparative animal models, including mice, ferrets, hamsters, and non-human primates (NHPs), that are being used to study clinical and pathological changes caused by influenza A virus infection with the aim to aid in appropriate model selection for disease modeling.

## 1. Introduction

The family *Orthomyxoviridae* encompasses four genera of influenza viruses: *Alphainfluenzavirus* (influenza A virus, IAV), *Betainfluenzavirus* (influenza B virus, IBV), *Gammainfluenzavirus* (influenza C virus, ICV), and *Deltainfluenzavirus* (influenza D virus, IDV) [1]. Influenza virus infections occur in people and animals worldwide and cause variable disease outcomes depending on the species affected and strain of virus. IAV and IAB infect humans and are responsible for seasonal influenza (flu) epidemics that result in 3–5 million severe illnesses and 290,000–650,000 deaths yearly worldwide [2]. Global flu pandemics, due to the introduction of new, antigenically distinct IAV strains in immunologically naïve populations, occur sporadically and cause increased morbidity and mortality [3]. IAV has a broad host range, infecting aquatic birds, domestic poultry, pigs, dogs, horses, bats, and people. Aquatic birds are considered the reservoir species and likely source of pandemic IAV in humans [4]. In contrast, IBV and ICV are primarily human pathogens, although IBV has been isolated from seals [5] and ICV from cattle [6], pigs [7], and dogs [8]. Infection with ICV is less common and mainly affects children [9]. IDV is a newly emerging virus detected in pigs [10] and cattle [11] with potential zoonotic risk [12]. 

**Figure 1:**
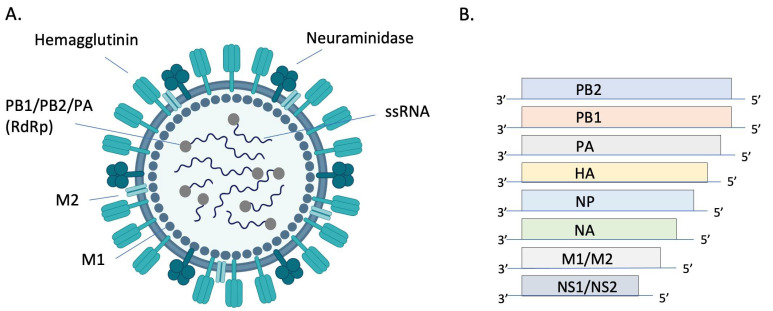
Structure and genomic organization of influenza A virus. The virion (**A**) consists of 8 negative sense ssRNA segments complexed with the nucleoprotein (NP, not pictured) to form the helical ribonucleoprotein (RNP). Each segment is associated with an RNA-dependent RNA polymerase (RdRP) complex composed of basic protein 1 (PB1), basic protein 2 (PB2), and the acidic protein (PA). Envelope-associated proteins include hemagglutinin (HA), neuraminidase (NA), matrix protein (M1), and the integral membrane protein (M2). Genomic segments 1–7 encode for one of these structural proteins and the 8th segment encodes for nonstructural proteins (NS) (**B**). Image (**A**) created with BioRender.com.

Orthomyxoviruses are enveloped, segmented, single-stranded, negative-sense RNA viruses. There are either 8 (IAV and IBV) or 7 (ICV and IDV) genome segments (Figure 1). The envelope of IAV and IBV is studded with the transmembrane glycoproteins hemagglutinin (HA) and neuraminidase (NA), which are responsible for attachment/penetration and virion release, respectively. IAVs are classified into subtypes based on their HA and NA molecules, of which there are 18 (HA) and 11 (NA) currently identified. All but two subtypes of IAV are found in aquatic birds while only H1N1 and H3N2 currently circulate in humans [13]. HA and NA are immunodominant epitopes with HA serving as the major target of neutralizing antibodies [14]. Random point mutations in HA and NA, due to the error-prone RNA polymerase, cause slow and gradual antigenic changes (antigenic drift) whereas genetic reassortment via exchange of gene segments results in new, antigenically distinct strains of virus (antigenic shift). These strategies allow the virus to evade host immune responses and develop resistance to antivirals. The possibility of zoonotic transmission and emergence of new pathogenic influenza strains of pandemic potential poses a significant public health threat.

In addition to the expected annual flu burden, pandemic influenza viruses have emerged every 10–40 years due to the reassortment of human IAVs with gene segments of avian and/or swine origin [15]. These novel viruses result in increased morbidity and mortality due to lack of preexisting immunity in human populations [16]. The 1918 H1N1 “Spanish flu” was the deadliest pandemic in history, causing over 40 million deaths worldwide [17]. In addition to the human influenza viruses causing seasonal and pandemic disease, people are sporadically infected with highly pathogenic avian influenza (HPAI) viruses of the H5 or H7 subtype, resulting in approximately 1000 deaths to date [18] and mortality rates of up to 60% [19]. 

Influenza viruses are transmitted by inhalation of droplets and/or aerosols and by direct contact with an infected individual or contaminated surface [20]. Prevention strategies include nonpharmaceutical interventions such as hand washing, use of hand sanitizer [21,22], social distancing, cough and sneeze hygiene, and cleaning of potentially contaminated surfaces [3]. Vaccination remains the most effective way of preventing disease. Efficacy of inactivated and live attenuated vaccines typically varies between 40–60% [23] depending on the age and health status of an individual, virulence of the season’s major strains of virus, and how well that season’s vaccine matches circulating strains [3]. Antivirals play an important role in combating disease, although drug resistance is a major challenge. Continued research efforts to develop universal influenza vaccines and novel antivirals are essential to combat this highly infectious and potentially fatal disease. The purpose of this review is to highlight the major pathologic features of select influenza animal models, including mice, ferrets, hamsters, and NHPs, with the ultimate goal of informing animal selection for disease modeling.

## 2. Human Disease and Clinical Symptoms caused by IAV and IBV

Most influenza infections are due to seasonal epidemics of IAV and IAB. Seasonal flu is typically an acute upper respiratory illness lasting 2 to 7 days [24]. Infection is often asymptomatic or a mild upper respiratory illness, although many people experience classic flu symptoms of varying severity, including fever, nonproductive cough, coryza, sore throat, fatigue, and myalgia [25]. While the acute illness typically abates within a week, coughing may continue for 2 or more weeks. Gastrointestinal symptoms such as vomiting, diarrhea, and abdominal pain can occur in severe disease and especially in children [26]. Infants, the elderly, and individuals with underlying health conditions are predisposed to severe infections and pneumonia is the most common complication leading to hospitalization. Other reported complications include myositis, myocarditis, encephalopathy, encephalitis, and Guillain-Barre syndrome [27]. Secondary bacterial pneumonia is more common than primary viral pneumonia [28,29]. Clinically, patients with secondary bacterial pneumonia will have an initial near recovery followed by recurrence of fever, cough, and dyspnea [27]. 

Pandemic flu causes a similar spectrum of disease ranging from mild to the classic flu-like illness described previously [30]. An unusual feature of the 1918 H1N1 pandemic was the disproportionate number of otherwise young, healthy adults affected, likely due to the unique virulence of this strain, dysregulated host immune responses, and lack of immune memory in the 25–35-year-old age group [17,31]. The 2009 H1N1 pandemic also caused more serious illness in children and adults under 65, although the overall case fatality rate was much lower than the 1918 pandemic [30]. Deaths during the 1918 H1N1 [28] and the 1957 H2N2 [32] pandemics can mostly be attributed to secondary bacterial infections with common upper respiratory tract pathogens. In contrast, bacterial coinfections were identified in only 26–38% of patients dying during the 2009 H1N1 pandemic with the majority of cases consisting of primary viral pneumonia and multiple organ failure [33,34]. 

Patients infected with zoonotic IAVs, such as HPAI H5N1 and H7N9 typically present with febrile flu symptoms and radiographic evidence of pneumonia following exposure to infected poultry [19,35]. Gastrointestinal symptoms are frequent in some reports [36,37]. Bacterial coinfections are uncommon in H5N1 infections [36,37,38] where death in severe cases occurs due to rapid respiratory failure and complications like acute respiratory distress syndrome and multiorgan failure [19]. 

## 3. Influenza Pathology

Influenza viruses enter host cells by binding of HA to sialic acid residues on glycoproteins and glycolipids. Binding affinity for specific sialic acid residues is an important determinant of host range, tissue tropism, and the potential for cross-species transmission (recently reviewed in ref [39]). In short, human influenza viruses preferentially bind to sialic acids with an α2,6 linkage to galactose (SAα2,6Gal) whereas avian influenza viruses preferentially bind to α2,3 linkages (SAα2,3Gal) [40]. In people, SAα2,6Gal is found predominantly in the upper airway and SAα2,3Gal mainly in the lower airway [41]. In contrast, birds express SAα2,3Gal predominantly in the upper airway and intestinal tract [42]. Thus, mutations that allow avian influenza viruses to bind to SAα2,6Gal in the upper respiratory tract of people are likely required for efficient human-to-human transmission via respiratory droplets and aerosols.

Influenza viruses replicate in the nucleus of respiratory and intestinal epithelial cells. Replication peaks at 48 h after infection and virus is shed for approximately 6–8 days [43]. Severity of disease is associated with viral replication in the lower respiratory tract [25]. While symptoms result from a combination of virus-mediated damage to epithelial cells and host immune responses (immunopathology), it is generally accepted that immunopathology plays the largest role in tissue damage [44]. Infected epithelial cells and innate immune cells release pro-inflammatory cytokines and chemokines that are important to control infection, but also lead to bystander damage to epithelial and endothelial cells. Excess neutrophil recruitment [45,46] and inflammatory cytokines such as IL-6, IL-8, IL-10, TNFα, CXCL10, IL-2R, GCSF, MCP1, and MIP1α are associated with disease severity and poor outcomes [35,47,48,49].

Because lung specimens are usually collected during autopsy, pathology is only documented in fatal cases of human IAV infection. Characteristic findings of viral pneumonia are similar between pandemic and non-pandemic years [25,32,34,50,51,52,53,54] so are described together. Grossly, the trachea and bronchi are hemorrhagic and often filled with blood-tinged, foamy fluid. The lungs are dark red and edematous, reflecting the underlying hemorrhagic bronchopneumonia that is frequently complicated by secondary bacterial infection [32,34,53]. Histologically, the trachea and bronchi have epithelial necrosis and desquamation in addition to submucosal edema, congestion, and hemorrhage. In the lower airways, there is necrotizing bronchiolitis and alveolitis, interstitial mononuclear inflammation, interstitial and alveolar edema, thrombi, hyaline membranes, and type II pneumocyte hyperplasia. These changes are consistent with the exudative phase of diffuse alveolar damage (DAD), which is the histologic hallmark of acute respiratory distress syndrome (ARDS) [55]. With time, epithelial regeneration, interstitial fibrosis, and bronchiolitis obliterans may develop. Secondary bacterial pneumonia consisting of overwhelming neutrophilic inflammation, extensive necrosis, and hemorrhage can obscure the underlying viral effects [25]. Hemophagocytosis is a prominent feature of some cases of fatal disease and is thought to be mediated by hypercytokinemia [33,51]. Hemophagocytosis was present in the lymph nodes of 18 of 36 (53%) pediatric patients dying of non-pandemic influenza [52] and in 25 of 41 (61%) patients dying of 2009 pH1N1 [33]. The clinical significance of hemophagocytosis in these infections is unclear.

While overall histologic findings are similar in fatal cases from pandemic and non-pandemic years (Table 1), antigen distribution appears to differ. In a study of 47 pediatric patients dying of seasonal influenza pneumonia from 2003–2004, antigen was detected primarily in the bronchial epithelial cells and mucous glands of the trachea, bronchi, and large bronchioles [52]. In contrast, antigen was mainly present in type I and II alveolar pneumocytes in patients dying of 2009 pandemic H1N1 (pH1N1) [33]. These findings likely reflect differences in tissue tropism based on receptor location and the variable course of disease at the time of sampling. 

As the case numbers are significantly lower compared with seasonal and pandemic H1N1, there are fewer reports on the pathology of fatal avian H5N1 infection despite a case fatality rate of 56% [56]. Diffuse alveolar damage is the main histologic feature and most cases also display mild lymphocytic interstitial pneumonia, alveolar histiocytosis, hemorrhage, and type II pneumocyte hyperplasia [51,57,58,59,60,61,62,63,64]. Depending on the time course of disease, DAD may be exudative or organizing and fibrotic [62,63]. Extrapulmonary lesions including lymphoid depletion, hepatic necrosis, and acute tubular necrosis, are frequently reported [51,58,60,61,65]. Viral RNA can be detected outside of the respiratory tract in the spleen, liver, intestines [62,63,64], and cerebrospinal fluid [66] although extrapulmonary antigen is only rarely documented [61], suggesting that systemic spread may not be the culprit of multiorgan failure. In the respiratory tract, viral antigen and RNA are found most commonly in type I and II pneumocytes although they can also be present in macrophages, sloughed epithelial cells, non-ciliated and ciliated bronchiolar epithelium, and tracheal epithelium [57,58,60,61,62,63]. The predominant viral distribution in pneumocytes is consistent with the lower airway distribution of SAα2,3Gal, the receptor for avian influenza viruses [41]. Hemophagocytosis is a frequent finding in the lungs, lymph nodes, and spleen [51,57,58,60,61,63,64]. In severe cases, reactive hemophagocytic syndrome, consisting of pancytopenia, abnormal clotting times, and reduced liver function, is the most prominent finding [51,60]. A combination of high viral loads and an intense cytokine response appear central to the pathogenicity of H5N1 in people [57,67,68,69].

**Table 1:** Summary of clinical and pathologic findings in human and laboratory animal influenza infections.

	Common Animal Strain/Species	Virus Strain	Clinical Signs	Microscopic Pathology	References
**Humans**	N/A	Seasonal IAV	Varying degrees of fever, non-productive cough, dyspnea, coryza, fatigue, and myalgia (classic flu symptoms); vomiting and diarrhea in severe cases	Necrotizing tracheobronchitis and bronchointerstitial pneumonia with thrombi, edema, hemorrhage, hyaline membranes, and type II pneumocyte hyperplasia (diffuse alveolar damage)	[25,26,50]
		2009 pH1N1	Mild to severe flu symptoms	Same as seasonal IAV	[30,34,50,52]
		1918 H1N1	Mild to severe flu symptoms	Same as seasonal IAV	[17,25,53]
		HPAI H5N1 and H7N9	Mild to severe flu symptoms with history of contact with live poultry	Same as seasonal IAV Extrapulmonary necrotic lesions are common and hemophagocytosis may be the most prominent lesion	[19,35,36,37,50,51,70,71]
**Mouse**	BALB/c C57BL/6 DBA/2J A/J	PR8	Dyspnea, ruffled fur, weight loss, and anorexia	Interstitial pneumonia, suppurative bronchiolitis and alveolitis, hyaline membranes, and alveolar edema	[72,73,74,75]
	BALB/c C57BL/6	2009 pH1N1	Variable weight loss (dose and strain dependent)	Histiocytic to neutrophilic bronchitis, bronchiolitis and alveolitis with varying epithelial necrosis (strain dependent)	[76,77,78,79]
	BALB/c	1918 H1N1	Weight loss and death	Interstitial pneumonia, suppurative bronchiolitis and alveolitis, hyaline membranes, and alveolar edema	[76,80,81]
	BALB/c DBA/2J	HPAI H5N1	Dyspnea, ruffled fur, weight loss, and anorexia	Interstitial pneumonia, suppurative bronchiolitis and alveolitis, hyaline membranes, and alveolar edema Encephalitis and myocardial necrosis	[81,82,83,84,85,86,87,88]
	C57BL/6 BALB/c	H7N9	Weight loss, ruffled fur, hunching	Bronchiolitis, patchy interstitial pneumonia, and varying amounts of bronchiolar and alveolar epithelial necrosis	[89,90,91,92]
	BALB/c	LPAI	Variable weight loss, ruffled fur, and hunching (strain dependent)	Necrotizing bronchitis and bronchiolitis with peribronchial pneumonia	[93,94,95,96,97]
**Hamster**	Golden Syrian hamster	Seasonal IAV	Mild weight loss and temperature changes	None or mild necrotizing rhinitis and bronchopneumonia with perivascular cuffing	[98,99,100,101,102]
		2009 pH1N1	Mild weight loss	Necrotizing rhinitis, bronchiolitis, perivasculitis, edema, and mild interstitial pneumonia	[101,103,104,105]
		HPAI H5N1	Not reported	Intranasal route: Bronchiolitis and bronchopneumonia Intragastric route: Interstitial pneumonia	[106]
**Ferret**	*Mustela putorius furo*	Seasonal IAV	Asymptomatic or mild lethargy with sneezing, nasal discharge, and mild weight loss	Conventional intranasal model: Rhinitis and mild bronchiolitis and pneumoniaHigh dose intratracheal model: Moderate rhinitis and severe necrotizing bronchointerstitial pneumonia with edema	[107,108,109,110,111,112]
		2009 pH1N1	Lethargy, anorexia, dyspnea, and elevated body temperature	Necrotizing rhinotracheitis, bronchitis, and bronchiolitis with varying degrees of interstitial pneumonia and diffuse alveolar damage	[77,110,111,112]
		1918 H1N1	Weight loss, sneezing, dyspnea, lethargy, and death	Necrotizing rhinitis, bronchiolitis and bronchointerstitial pneumonia with edema	[113,114,115]
		HPAI H5N1	Lethargy, anorexia, dyspnea, nasal discharge, sneezing, weight loss, elevated body temperature, diarrhea, and neurologic signs (ataxia, torticollis, and hind limb paresis)	Severe necrotizing bronchointerstitial pneumonia with diffuse alveolar damage Meningoencephalitis	[111,116,117,118,119]
		LPAI	Transiently elevated body temperature and weight loss; occasional dyspnea and lethargy	Suppurative rhinitis	[91,120,121]
**NHP**	Cynomolgus macaques (most common), rhesus macaques, and common marmosets	Seasonal IAV	Asymptomatic or mild lethargy	Mild bronchointerstitial pneumonia and peribronchiolitis	[122,123]
		2009 pH1N1	Asymptomatic or mildly elevated body temperature and lethargy; tachypnea, dyspnea, and nasal discharge reported for some strains of virus	Suppurative rhinitis in mild cases; necrotizing bronchopneumonia with edema and hyaline membranes in severe cases (strain dependent)	[124,125,126,127,128,129]
		1918 H1N1	Anorexia, lethargy, cough, nasal discharge, and tachypnea	Necrotizing bronchointerstitial pneumonia with hemorrhage, edema, and hyaline membranes	[123,130,131]
		HPAI H5N1	Anorexia, lethargy, cough, tachypnea, elevated body temperature, diarrhea, and thrombocytopenia	Necrotizing bronchointerstitial pneumonia with hemorrhage, edema, hyaline membranes, and type II pneumocyte hyperplasia Lymphoid necrosis and renal tubular necrosis	[130,132,133,134]

## 4. Comparative Pathology of Animal Models for Influenza A 

### 4.1. Mouse Models

Mice (*Mus musculus*) are the most common laboratory animal model of influenza. Benefits of using mice include their cost efficiency and the abundant immunologic and genetic tools available. Laboratory mice, however, are not susceptible to infection with many wild-type human influenza viruses but are readily infected with mouse adapted strains generated via serial passage through mouse lung [135,136]. The most common mouse strains used for influenza research are C57BL/6 and BALB/c, although DBA/2J and A/J mice are more susceptible to disease [72,73]. Susceptible inbred strains have a large deletion or nonsense mutation in the interferon inducible *Mx1* gene and thus fail to express the influenza restriction factor Mx1 [137]. Wild mice, in contrast, resist infection with even mouse adapted strains due to a functional *Mx1* gene [138]. Pathogenesis studies in C57BL/6 and BALB/c mice must therefore be interpreted with caution, especially regarding innate anti-influenza immunity. 

**Figure 2:**
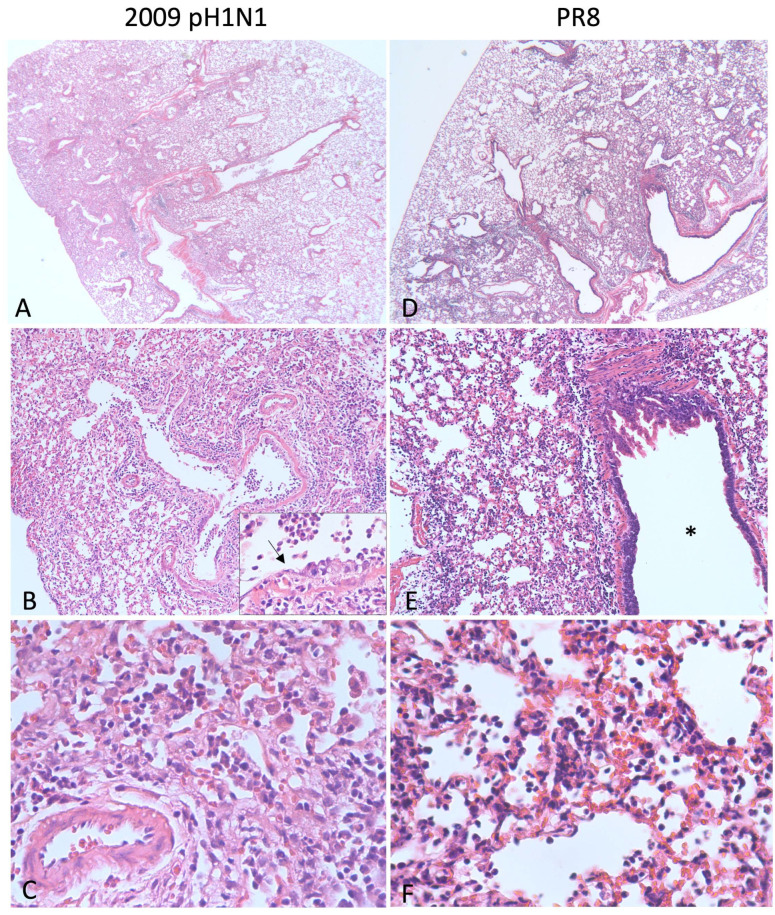
Lung pathology in mice infected with influenza. (**A**–**C**) BALB/c mouse inoculated with 1000 plaque forming units (pfu) of 2009 pH1N1 intranasally and euthanized on day 5. At low magnification (**A**), approximately 30% of the lung is consolidated and hypercellular. There is degeneration, necrosis, and loss of the bronchiolar epithelium (**B**) and a neutrophilic exudate (inset, arrow = degenerating epithelium). Alveolar septa are expanded by fibrin, edema, hemorrhage, neutrophils, lymphocytes, and plasma cells and alveolar spaces are filled with fibrin and degenerate neutrophils (**C**). (**D**–**F**) Tropomyosin receptor kinase A-knockin mouse (C57BL/6 background) inoculated with 250 pfu PR8 and euthanized on day 5. Similar to 2009 pH1N1, approximately 30% of the lung is consolidated (**D**). The inflammation affects the alveoli, and the airway (*) is spared (**E**). Alveolar septa are thickened due to congestion and the accumulation of neutrophils, lymphocytes, and macrophages. Alveolar spaces contain neutrophils, necrotic debris, and fibrin (**F**).

The commonly used laboratory adapted influenza A/Puerto Rico/8/34 strain (PR8) is lethal in C57BL/6 [72] and BALB/c mice [74,75]. Clinical signs include tachypnea, ruffled fur, weight loss, and anorexia. Grossly, the lungs are dark red and consolidated [74]. Infected mice have characteristic viral pneumonia consisting of interstitial inflammation and neutrophilic bronchiolar exudates [72,74]. Examples of histologic lesions are shown in Figure 2. Fukushi et al. [74] compared the pulmonary lesions of BALB/c mice surviving infection with those that died or were humanely euthanized and found that dead mice had characteristic lesions of DAD, including hyaline membranes, edema, and alveolar collapse, whereas as survivors did not. As previously described, DAD is the typical histologic finding in fatal cases of influenza viral pneumonia in people (Table 1), so the PR8 mouse model can be used to test vaccines and therapeutics [75,139] and to investigate pathogenesis of severe disease [140,141] with the notable caveat that the virus is adapted to mice.

Mice are susceptible to certain human IAV strains without prior adaptation. The reconstructed 1918 H1N1 influenza virus is lethal in BALB/c mice where it causes necrotizing bronchitis, bronchiolitis, and neutrophilic alveolitis with alveolar edema and hemorrhage [80] similar to patients infected in 1918 [53]. By infecting mice with the fully reconstructed recombinant 1918 H1N1 virus or other recombinant viruses containing one or more 1918 H1N1 genes, Tumpey et al. [80] showed that the HA and polymerase genes likely played a large role in the unusual lethality of the pandemic. The 2009 pH1N1 virus also replicates efficiently in mouse lungs without prior adaptation. Weight loss and pathology are both dose- and strain-dependent and generally non-lethal [76,77,78,79]. The histologic lesions of 2009 pH1N1 (strain California/4/09) are shown in Figure 2A–C and include necrotizing bronchiolitis and interstitial pneumonia. Pneumonia due to the mouse-adapted H1N1 virus PR8 is similar in this case, although the airways are relatively spared (Figure 2D–F). 

Because mice mainly express SAα2,3Gal rather than SAα2,6Gal on airway epithelial cells and type II pneumocytes [142], they are readily infected with avian IAVs. Avian IAVs attach predominantly to ciliated and non-ciliated cells in the trachea with lesser attachment in the lower respiratory tract of birds [143]. This pattern is opposite to avian IAV attachment patterns in humans, where virus mainly attaches to alveolar macrophages, type II pneumocytes, and nonciliated bronchiolar epithelial cells of the lower respiratory tract [143,144]. Common laboratory mouse strains are susceptible to the non-adapted HPAI strains H5N1 [82,83,84,85,86] and H7N9 [89,90] as well as some strains of low pathogenicity avian influenza (LPAI) [93]. Mice infected with HPAI H5N1 exhibit rapid weight loss and clinical signs such as hunched posture, ruffled fur, inappetence, and dyspnea within 24 h of infection [83,84,86]. Clinical signs, lesions, and lethality are dependent on the virus strain, with strains originating from the Hong Kong outbreak in 1997 causing the most severe disease [82,86]. In lethal infections, there is degeneration and necrosis of epithelial cells lining the nasal cavity, trachea, bronchi, and bronchioles with associated intraluminal fibrinosuppurative inflammation. Alveolitis typically occurs in a peribronchial pattern and consists of pneumocyte necrosis and accumulations of fibrin, erythrocytes, neutrophils, and increased alveolar macrophages in alveolar spaces [86]. Antigen is found in respiratory epithelial cells of the nasal cavity, bronchi, and bronchioles, and within sloughed necrotic cells [82,84,86]. In addition to pneumonia, some have shown cardiomyocyte necrosis [83] and encephalitis [84,87] with accompanying influenza viral antigen, indicating systemic infection. The neurovirulent influenza mouse model is particularly relevant because encephalitis is a known, although uncommon, complication of H5N1 infection [66,145]. H7N9 is generally less pathogenic than H5N1 in mice, causing weight loss, multifocal peribronchial interstitial pneumonia, and variable necrosis of bronchiolar and alveolar epithelia [89,90,91,92]. 

Mice are an efficient and low-cost influenza model that has been indispensable for the discovery of new vaccines (reviewed in ref [146]) and therapeutics [147,148,149] and gaining insight into disease pathogenesis (Table 2) [80,84,87,141]. A major drawback of this model is that mice are not naturally susceptible to many strains of human IAV. They are, however, susceptible to mouse-adapted and certain highly pathogenic human and avian IAV strains. Additionally, transmission is generally inefficient in this species [150,151]. Additional animal models described in the following sections serve to fill these gaps.

### 4.2. Ferret Model

Ferrets (*Mustela putorius furo*) were the first animal experimentally infected with influenza virus [160] and are still the one of the best models of infection [161]. They are naturally susceptible to seasonal human influenza isolates [107,108], 1918 and 2009 pandemic H1N1 influenza [84,117,120,121], LPAI [91,120], HPAI [116,117,118] and other animal-derived influenza viruses [121,162]. Ferrets exhibit upper respiratory symptoms similar to humans such as sneezing, nasal discharge, lethargy, and fever [163] and transmit virus via respiratory droplets [116,155,164]. Patterns of transmission between ferrets are similar to those in humans, so transmission studies can help predict the potential of a novel influenza virus to cause a pandemic [154]. The distribution of sialic acids in the respiratory tract is similar between ferrets and humans [165,166], so, like in people, human influenza viruses attach to ciliated epithelial cells in the trachea and bronchi and type II pneumocytes in ferrets [143].

The severity of disease depends on dose, route of inoculation, and strain of IAV. With human seasonal IAVs, the conventional intranasal challenge model of 1 × 10^6^ median tissue culture infectious dose (TCID_50_) predominantly causes rhinitis and bronchiolitis [110,112] and mild, localized bronchointerstitial pneumonia [109]. Intratracheal inoculation is used to reliably induce lower respiratory tract inflammation [111,167,168]. High intratracheal doses (1 × 10^9^ TCID_50_) of seasonal influenza strains are required to cause reproducible necrotizing bronchointerstitial pneumonia [168]. In comparison, the 2009 pH1N1 virus causes diffuse alveolar damage and necrotizing pneumonia at standard challenge doses [111]. Ferrets inoculated intraocularly have similar antigen distribution and inflammation compared to those inoculated intranasally [169], representing another route of mucosal exposure [170].

Ferrets are also highly susceptible to infection with select agent IAVs, including HPAI H5N1 [111,119,167] and the reconstructed 1918 H1N1 influenza virus [113,114,115]. Tumpey et al. [113] used the ferret model to show the importance of HA receptor specificity in the transmission of pandemic viruses; reconstructed 1918 H1N1 viruses with mutations causing a change in binding preference from SAα2,6Gal to SAα2,3Gal lost the ability to be transmitted between ferrets. The viral RNA polymerase complex was identified by Watanabe et al. [115] as an essential virulence factor of 1918 H1N1 influenza in ferrets. These studies illustrate the importance of this model in understanding the pathogenesis of influenza viruses with a particular focus on those with pandemic potential.

HPAI H5N1 and 2009 pH1N1 infections in ferrets causes a range of clinical signs including lethargy, inappetence, dyspnea, sneezing, increased body temperature, and weight loss [111,119,167,171]. Seasonal IAV infections are often asymptomatic or may result in mildly reduced activity levels [111,119]. Of these strains, HPAI H5N1 causes the most severe clinical disease and pathology [111,119]. Extrarespiratory disease like diarrhea [119,171] and neurologic signs (e.g., ataxia, torticollis, and hind limb paresis) [119,167,171] are also common with H5N1 infection. The infection is often lethal, especially at high intratracheal doses [111]. Zitzow et al. [119] infected ferrets with two strains of H5N1 and found that both replicated at high titers in the lungs and nasal turbinates. Virus was also detected in the brain, spleen, intestine, and fecal swabs of some animals, consistent with systemic infection seen in people infected with H5N1 [69,172]. Grossly, infected animals have dark red, consolidated lungs [119,167] and histologic lesions are characterized by severe necrotizing bronchointerstitial pneumonia and diffuse alveolar damage [111,119,171]. Brain lesions consist of nonsuppurative and necrotizing meningoencephalitis with glial nodules and neuronophagia [119,167,171]. The route of inoculation plays a role in the severity of central nervous system and pulmonary lesions. Bodewes et al. [167] found that all ferrets inoculated intranasally with H5N1 had necrotizing encephalitis while fewer than half had mild to moderate bronchointerstitial pneumonia. In comparison, ferrets inoculated intratracheally with a lower dose were more ill and all had severe pneumonia, no neurologic symptoms, and five times greater viral titers in the lungs. They hypothesized that direct extension of virus from the nasal cavity to the olfactory bulb during intranasal inoculation led to viral encephalitis.

As shown here, ferrets are an invaluable tool for dissecting influenza virus transmission and pathogenesis (Table 2), especially in the face of potential future pandemic viruses. The anatomy and physiology of the ferret respiratory tract is similar to humans [173] and they develop pulmonary lesions that accurately reflect human disease (Table 1). Drawbacks of this model are increased costs, lack of inbred strains, and fewer available reagents when compared to mice [160].

### 4.3. Hamster Model

The Syrian (Golden) hamster (*Mesocricetus auratus*), like the ferret, is naturally susceptible to IBV [174] and many strains of IAV, including seasonal viruses [98,99,100,101], pandemic 2009 H1N1 [101,103,104], and avian H5N1 [106]. They are small, inexpensive, and are simpler to manage when compared to ferrets. Hamsters were first used to study immune responses to infection and immunization [100,175,176,177], although other small animal models subsequently became more popular. There has recently been renewed interest in the hamster influenza model, especially as a model for coronavirus and IAV coinfection [103,104,105,152].

IAV in hamsters is largely an upper respiratory tract infection. Virus is readily retrieved from nasal washes of infected animals [100,101,175,177] with titers peaking at 3–4 days post-infection (dpi) and clearing by 7 dpi [100,101]. Clinical signs that can be observed in ferrets, like nasal discharge, lethargy, and sneezing, have not been reported in hamsters regardless of the IAV strain [101,102,103,177]^.^ Mild weight loss may occur without other observable signs of infection following inoculation with pH1N1 [101,104] and seasonal H1N1 strains [102]. Descriptions of influenza pathology in hamsters are limited and vary depending on the study and virus strain. Iwatsuki-Horimoto et al. [101] compared the nasal pathology associated with seasonal H3N2, 2009 pH1N1, and IBV strains. Necrotizing rhinitis concentrated in the olfactory epithelium was identified in pH1N1-infected hamsters, but no lesions were identified in H3N2- or IBV-infected hamsters. Antigen (IAV NP) was detected in the upper respiratory tract of some hamsters infected with H3N2 and in both the upper and lower respiratory tracts of hamsters infected with pH1N1 and IBV. In hamsters infected with 2009 pH1N1, pulmonary lesions vary from bronchiolitis with luminal exudates, perivasculitis, with edema [103] to mononuclear interstitial pneumonia that may be very mild (Table 1) [104,105]. Antigen (IAV NP) is present in nasal epithelial cells in hamsters infected with H3N2 [101] and in nasal, tracheal, bronchiolar, and bronchial epithelial cells but infrequently in alveolar pneumocytes in hamsters infected with pH1N1 [101,103]. This contrasts with fatal pH1N1 infection in people where antigen is largely located in pneumocytes [33]. Paterson et al. [102] infected hamsters with a recent seasonal strain of H1N1 that caused more weight loss and respiratory lesions compared to other studies using the 2009 pH1N1 strain. These hamsters had necrotizing rhinitis, laryngitis, and tracheitis with acute, necrotizing bronchopneumonia and perivascular cuffing. Lastly, hamsters are susceptible to avian H5N1 infection via intranasal and intragastric routes where they develop bronchopneumonia and interstitial pneumonia, respectively [106].

Hamsters are a cost-efficient and easy-to-manage small animal model of influenza infection that do not require viral adaptation. Like ferrets, hamsters can transmit influenza [101,178] and develop comparable lesions and immune responses [102,106]. Additionally, hamsters have both SAα2,3Gal and SAα2,6Gal in the upper respiratory tract and, like people, SAα2,3Gal in the lower respiratory tract, supporting their use as a model for the human disease. A major drawback is that they do not develop clinical signs and lesion severity is variable, which could preclude their use in testing the efficacy of antivirals and vaccines.

### 4.4. Non-Human Primate Models

Non-human primates (NHPs) are popular animal models due to their close genetic relationship with humans. As they share common anatomic and immunologic features, they are generally excellent models for viral respiratory diseases, including influenza. Their use, however, is limited by ethical concerns, high husbandry costs, and lack of availability compared to rodents and small mammals. NHPs are naturally [179] and experimentally susceptible to human [123,126,180] and avian [180,181] IAVs.

Rhesus (*Macaca mulatta*) and cynomolgus (*Macaca fascicularis*) macaques are the most frequently used NHP in influenza research, although other species including African green monkeys (*Chlorocebus sebaeus*), pig-tailed macaques (*Macaca nemestrina*), squirrel monkeys (*Saimiri* spp.), and common marmosets (*Callithrix jacchus*) have also been studied [182]. When infected with seasonal strains, NHPs are typically asymptomatic or have mild disease [122,123,180]. Similarly, 2009 pH1N1 is often a subclinical infection in NHPs [124,125,126] although varying degrees of respiratory illness can occur [127,128,129], which likely depends on the viral strain, species of NHP, and route of infection. Cynomolgus are generally preferred over rhesus macaques as they have higher and more consistent viral titers in the respiratory tract and increased pulmonary pathology [129,183]. This difference can likely be attributed to the greater number of SAa2,6Gal receptors in the trachea and bronchioles in cynomolgus compared to rhesus macaques [129].

Clinical signs in macaques infected with 1918 H1N1 or avian IAVs include fever, anorexia, lethargy, tachypnea, and cough that is often fatal [123,132,133]. Diarrhea and thrombocytopenia, which have been reported in people [36], may also occur in H5N1 infected macaques [132]. H5N1 and 1918 H1N1 antigen can be detected as early as 12 h post infection in bronchiolar and alveolar epithelial cells [130] with histologic lesions that include hemorrhage, edema, and necrotizing bronchiolitis and alveolitis starting by at least 24 h after infection (Table 1) [123,130,132]. Interstitial inflammation is composed of neutrophils and macrophages [132,134]; hyaline membranes and extra-pulmonary necrotic lesions are occasionally reported [134]. In contrast to studies showing severe and often lethal disease due to 1918 H1N1 in macaques [123,130,131], it was recently reported that infection even with high doses causes minimal disease in rhesus macaques and dose-dependent, non-lethal disease in cynomolgus macaques [183]. These discrepancies could be due to differences in study design, genetics, or euthanasia criteria. Regardless, cynomolgus macaques are, to date, the best NHP model for these deadly influenza virus infections.

Due to lack of accessibility, ethical concerns, and high costs associated with housing and husbandry, NHPs are used less often than small animal models. Because they are genetically and anatomically more similar to humans, however, they are a valuable tool for examining pathologic and immunologic responses to influenza (Table 2) [123,124,125,130,132,156,157].

## 5. Conclusions

Influenza viruses have caused multiple pandemics in the last century, including the deadliest recorded pandemic of any etiology in 1918. While zoonotic influenza strains have had limited human-to-human transmission to date, a virus that gains the ability to be efficiently transmitted between people would be a serious global threat. Great strides have been made in developing antivirals and vaccines, although a universal influenza vaccine remains elusive. In this review, we have described the major clinical and pathologic findings of four popular animal models—mice, ferrets, hamsters, and NHPs—that are summarized in Table 1 to aid researchers in selecting the most relevant disease model.

Mice are a convenient and cost-effective option, although they are not susceptible to most human IAV strains and lack an important innate antiviral effector [137]. They are, however, susceptible to avian IAVs and 1918 H1N1 and, like people, develop necrotizing interstitial pneumonia [80,86]. The mouse adapted IAV strain PR8 elicits many histologic features of severe influenza pneumonia in people, including alveolitis, hyaline membrane formation, and alveolar edema that characterize diffuse alveolar damage, so is a convenient system in which to test vaccines and therapeutics. Of the four species discussed here, ferrets display symptoms, transmission dynamics, and pathologic features that are most similar to people. Unlike mice, they are naturally susceptible to human IAVs and exhibit upper respiratory symptoms like sneezing and nasal discharge [163]. Using high dose intratracheal inoculation, severe bronchointerstitial pneumonia can be induced with seasonal IAV strains, which is a unique feature of the ferret model [111,112,167]. In comparison, hamsters are susceptible to seasonal and pandemic IAV strains but are asymptomatic [101,102,103,177]. NHPs are also asymptomatic when infected with seasonal IAV [122,123,180] but have been particularly valuable in studying pandemic H1N1 and avian strains as they develop pulmonary pathology, including the formation of hyaline membranes, that is very similar to people [123,130,132]. However, their use is generally limited due to high costs, lack of availability, and ethical concerns. The prominent hemophagocytosis seen in both H1N1 and H5N1 infections has yet to be replicated in these animal models. In general, influenza pneumonia is more severe in people than in laboratory animals. A great deal of information has been learned about influenza viruses using these models and they will continue to be essential for the development of effective therapeutic and vaccine strategies to combat potential emerging viruses.

## Figures and Tables

**Table 2:** Benefits, drawbacks, and main applications of animal models of influenza.

	Benefits	Drawbacks	Main Applications [Refs]
**Mouse**	Low cost Many tools available Genetically malleable Rapid rate of reproduction	Require adapted strains to model seasonal flu Lack influenza restriction factor Mx1 Do not sneeze and thus cannot model airborne transmission	Vaccine and therapeutic testing [75,139,152] Pathogenesis of severe disease [77,80,140,141]
**Hamster**	Low cost Simple management Naturally susceptible to human and animal IAVs Contact transmission Similar sialic acid residue distribution to humans	Fewer reagents Lack of clinical signs like sneezing and nasal discharge Not very friendly	IAV and coronavirus co-infection [103,104,105,153]
**Ferret**	Naturally susceptible to human and animal IAVs Transmission via respiratory droplets Similar sialic acid residue distribution to humans	Fewer reagents Lack of inbred strains Increased cost	Transmission studies [113,116,154,155] Pathogenesis [115]
**NHP**	Anatomic and immunologic similarities to humans Naturally susceptible to human and animal IAVs	High cost Complicated husbandry requirements Ethical concerns Lack of availability	Immunology [123,124,125,130,132,156,157] Vaccine and therapeutic testing [131,133,158,159]

## Data Availability

No new data were created or analyzed in this study. Data sharing is not applicable to this article.
